# Cervical and Corporeal Endo-metritis, with Retroflexion

**Published:** 1869-12

**Authors:** F. B. Norcom

**Affiliations:** Chicago, Ill.


ARTICLE VIII.— *Cervical and Corporeal Endo-Metritis, with Retro-flexion.* By F. B. NORCOM, M.D., Chicago, Ill.

ON 25th of January of this year I was summoned to visit a young lady, aged 19 years, of nervous temperament, who had been suffering the last 18 months from profuse and painful menstruation and general bad health. Her monthly periods began about her sixteenth year, were always regular and painless, and her condition that of excellent health, up to one year and a half ago, when she suffered from an attack of amenorrhœa, which continued three months, to be followed by a severe uterine hæmorrhage, attended by pains in loins, pelvis, and lower abdomen, with expulsion of large clots of blood. Two months afterwards she accidentally fell down a stairway, bruised herself, and ever since her courses have been dysmenorrhœal, at times very profuse, and accompanied and followed by pelvic, abdominal, and lumbar pains. During the intervals slight leucorrhœa, a feeling of fulness in uterine region, pretty constant — but not severe — pains, and gradual failing of her ordinary health.

Miss K—— presented at first sight, a pale, rather haggard expression, highly strong nervous tendency, coated tongue, and weak, irritable pulse, about 90. She suffered much from gastric distress and fullness, especially after meals, having little or no appetite, and was much troubled with broken sleep and frightful dreams. She complained of great weakness, and such pain in her back that, she could in no wise attend her household duties. And although she had submitted to constant medical treatment, both local and general, she felt no better, and had fallen into despondency and disgust.

An examination by the *toucher* revealed a thickened cervix, with open os, retroflexion and much tenderness with some hypertrophy of body of uterus. The sound passed readily in the reverse position, at least three inches, giving acute pain upon movement in the uterine cavity, and by turning easily replacing the womb in its natural position, but which as readily returned, very much to her immediate relief. The os tincæ upon view appeared red and swollen, the canal well opened, its membrane slightly everted and granular, while a clear, glossy mucus exuded, and fell into the vaginal canal.

My opinion was, that there had been abortion, followed by its very ordinary subsequences, inflammation of membrane of neck and cavity, subsequently, of the body, and displacement from accident. Scarifications and warm vaginal douches were resorted to, while the bowels were kept soluble by alkaline cathartics, and vegetable tonics and bismuth, with very slight percentage of morphine for dyspeptic symptoms. About one week after this initiatory treatment the ordinary sponge tent was introduced late in the afternoon, and renewed next morning, when by proper manipulation the cervical canal was exposed to view, and presented the condition of granular inflammation so common to its lining membrane. An application of chromic acid — ʒ j to ʒ ij — was then made, the neck painted over with Churchill's strong tincture of iodine, and the posterior *cul-de-sac* well filled with iodized cotton moistened in glycerine. The tent was used once a week, always followed by the chromic acid solution, and in the interval reposition was daily effected, cold and warm injections assiduously applied, hip bathing, proper decubitus in bed, liberal diet, regulated exercise, etc., etc.

About the middle of March, the tents having been discontinued several weeks, there was such amelioration of the inflammatory symptoms, such as pain, tenderness, weight, etc., that I inserted, in order to facilitate the cure, the closed bar pessary, and the womb immediately took position in its natural bed. This support remained in until the latter part of April, when she complained of its giving rise to pain in left side of vagina, and producing irritation of neck of bladder. It was removed, warmed over a spirit lamp and properly moulded, by slightly curving the left lateral bar, bringing forward its posterior support, and indenting and lowering its anterior bar, so that when again replaced, it seemed to fit, or be exactly adapted to the condition of the parts, and was worn until August, when I considered the cure as complete, and removed it to find the uterus in its normal position well retained in the pelvis. Her appetite had now returned, her sleep refreshing, all pain, of whatever character, gone, her spirits and buoyancy of disposition regained. Her menstruation had been gradually assuming its natural condition, both as regards quantity and quality, and at date of the removal of support was perfect.

In this case reposition could have been effected without the pessary, but it would have taken probably more time, and it was used simply to facilitate a cure, not *ex necessitate rei*.

